# Non‐traditional mental health clinical placements: An effective means for reducing self‐stigma in pre‐registration nursing students

**DOI:** 10.1111/jpm.13093

**Published:** 2024-08-08

**Authors:** Christopher Patterson, Michelle Roberts, Taylor Yousiph, Georgia Robson, Kelly Lewer, Elissa‐Kate Jay, Lorna Moxham

**Affiliations:** ^1^ Faculty of Science, Medicine and Health University of Wollongong Wollongong New South Wales Australia

**Keywords:** clinical placement, mental health, nursing education, self‐stigmatizing attitudes, stigma

## Abstract

**What Is Known on the Subject:**

- Pre‐registration nursing students report high rates of stigma, leading to low help‐seeking attitudes when seeking help for mental health issues.
- Traditional mental health clinical placements can improve stigma related to attitudes and social distance for pre‐registration nursing students.

**What the Paper Adds to Existing Knowledge:**

- There are nil recorded clinical placement interventions that have decreased self‐stigma for pre‐registration nursing students, with this study highlighting a clinical placement model that is effective in significantly decreasing self‐stigma.

**Implications for Practice:**

- The knowledge around the mental health struggles experienced by pre‐registration nursing students, and the effect of a non‐traditional mental health placement in decreasing self‐stigmatizing attitudes in this population, is important for the future of retaining mental health nurses.
- There is an opportunity to use the clinical placement model presented, and design interventions for nursing students that aims to promote help‐seeking behaviours.

**Abstract:**

**Introduction:**

Traditional mental health clinical placements can improve pre‐registration nurse stigma toward mental illness, particularly in measures of attitudes and social distance. However, they have not yet been shown to improve self‐stigma, which affects mental health disclosure and help‐seeking behaviour.

**Aim:**

The present study investigates nursing students' stigma following a non‐traditional mental health placement immersed alongside people living with mental illness.

**Methods:**

Three stigma subtypes were measured using the Opening Minds Scale for Healthcare Providers: *Attitudes*, *Social Distance*, and *Disclosure/Help‐seeking*.

**Results:**

Pre‐registration nurses (*N* = 848) completed the instrument pre‐ and post‐placement. A multivariate analysis of variance (MANOVA) identified a large effect of placement on stigma (*p* < .001, $\eta_{p}^{2}$= .101). Post hoc pairwise comparisons revealed all three types of stigma decreased after the non‐traditional placement (*Attitudes*: *p* < .001, $\eta_{p}^{2}$= 0.09, *Social Distance: p* < .001, $\eta_{p}^{2}$= 0.07, *Disclosure/Help‐seeking*: *p* < .001, $\eta_{p}^{2}$= 0.04).

**Discussion:**

These findings emphasize that attending a non‐traditional mental health clinical placement can effectively reduce multiple types of nursing student stigma.

**Limitations:**

Further research in this area could focus on which attributes of the clinical placement setting foster positive help‐seeking.

**Implications:**

These results are noteworthy for stigma surrounding disclosure/help‐seeking, as traditional (i.e. hospital‐based) mental‐health clinical placements have been found ineffective in reducing nursing student stigma in this domain.

**Recommendations:**

Further research into the effectiveness of non‐traditional clinical placements in reducing nursing students' stigma regarding mental health disclosure and help‐seeking, is required.

## 1 INTRODUCTION

Mental health nurses are the largest cohort of mental health workers. Their role is vital in assisting individuals in their personal recovery (World Health Organization, 2022a; 2022b). However, a critical shortage of these nurses exists (Productivity Commission, 2020). Furthermore, mental health nursing is the least preferred specialty when students commence their registered nurse career (Lim et al., 2020). Stigma is thought to be a barrier contributing to fewer nursing students' intention to practice mental health nursing (Thongpriwan et al., 2015). This could be a consequence of the lack of theoretical education and effective mental health clinical placements to facilitate the interaction of nursing students with people living with mental illness (Happell & Gaskin, 2013).

### 1.1 Mental health of nursing students

The mental health of nursing students is a concern recognized worldwide (Reverté‐Villarroya et al., 2021). The combination of being both a health professional and student places considerable stress on pre‐registration student nurses (Hirani et al., 2021; Hughes et al., 2019). It is well documented that nursing students experience high levels of emotional distress, attributed to academic pressure, stress surrounding clinical placement experiences, and psychosocial stressors (Deasy et al., 2016; Thomas & Revell, 2016). In a meta‐analysis of studies investigating depression rates in nursing students, it was found that globally, 34% of nursing students experience mild to severe depression (Tung et al., 2018). Recently, and in the wake of the COVID‐19 pandemic, a study from Israel found 42.8% of undergraduate nursing students have reported moderate anxiety (Savitsky et al., 2020) and found that while depression in nursing students was of concern, there was also an increase in anxiety levels (Black Thomas, 2022; Mosteiro‐Diaz et al., 2023). While the burden of academic stress is high for nursing students, symptoms of anxiety and depression reduce as students advance in their studies, potentially related to the development of stress‐management strategies over the course of their academic studies or because of increased confidence in clinical material (Reverté‐Villarroya et al., 2021).

Undergraduate nurses are the future of the healthcare workforce, and understanding needs relating to their mental well‐being, particularly with the effects of COVID‐19 (Moxham et al., 2022), is crucial (Hirani et al., 2021). Addressing the mental health of nursing students and implementing interventions to support them is critical. This is markedly so amidst the underserved mental health needs and ongoing stigma related to mental health for healthcare providers (Hirani et al., 2021; Sonmez et al., 2023). Recommendations including the need for nurse educators to provide additional support for undergraduate nurses in the development of coping skills, providing education on stress management, and giving opportunities for self‐care including mindfulness exercises, could be beneficial (Aloufi et al., 2021). Compounding this issue, however, are low help‐seeking behaviours amongst nursing students, and high levels of self‐stigma, resulting in a reluctance to seek mental health support which can intensify psychological distress (Deasy et al., 2016; Foster et al., 2019).

### 1.2 Stigma in nursing students

Mental health stigma is defined as the ‘disgrace, social disapproval, or social discrediting of individuals with a mental problem’ (Subu et al., 2021, p. 2). Negative opinions or judgements about mental illness can foster stigma, and subsequently lead to feeling isolated, low self‐esteem, and sometimes, suicidal thoughts (Chang et al., 2017; New South Wales Health, 2020). People living with mental illness often feel dismissed and dehumanized by health professionals due to stigma (Hamilton et al., 2016). Some of the main sources of stigma in healthcare include negative attitudes and behaviours, lack of awareness, therapeutic pessimism, lack of skills related to mental health care and stigmatizing attitudes in workplace culture (Knaak et al., 2017). Stigmatizing attitudes of nurses specifically result in compromised person‐centred care and a negative nurse‐client relationship (Tyerman et al., 2021).

The negative impact of stigma within the healthcare system for people living with mental illness, has prompted a targeted focus on nursing education aiming to reduce stigmatizing attitudes (Knaak et al., 2017; Productivity Commission, 2020). Currently, nursing students show a negative attitude toward mental illness (Meng et al., 2022) but factors that can positively influence nursing students' level of stigma include, a higher education level (Meng et al., 2022), personal exposure (Foster et al., 2019; Hawthorne et al., 2020) and older age (Hsiao et al., 2015). The necessity for interventions in nursing education to reduce stigma is crucial amidst the need to recruit mental health nurses and the rising mental health needs of the population. Interventions focussed on the involvement of people with lived experience in the nursing curriculum is promising (Happell et al., 2018), alongside exposure to consumers in a clinical placement experience (Foster et al., 2019). Traditional mental health placements have not been as effective in reducing attitudes to stigma compared to alternative mental health placements (Perlman et al., 2020). Nor, by comparison, have they effectively reduced social distance; that is, the extent to which someone is willing to participate and engage in social contact with a person with a mental illness. Chang et al. (2017) however, found that while healthcare students have positive attitudes toward help‐seeking for mental health consumers, they are not comfortable disclosing their own mental health condition or distress.

### 1.3 Self‐stigma

Stigma is a multifaceted phenomenon, and can consist of intrapersonal or self‐stigma, interpersonal (relations with others) and structural stigma (discriminatory systems or policies) (Knaak et al., 2017; Meng et al., 2022). Self‐stigma is the internalization of negative public attitudes toward mental illness and the experience of negative consequences as a result (Corrigan & Rao, 2012). Self‐stigma encompasses three main elements including stereotype, prejudice, and discrimination, and refers to the stigma expressed toward oneself, not others (Gärtner et al., 2022). While public stigma may result in the avoidance of seeking help for people with mental illness, self‐stigma affects feelings of self‐esteem and self‐efficacy, hindering disclosure and help‐seeking behaviours (Pattyn et al., 2014). Self‐stigma may further lead to feelings of guilt and shame, contributing to social withdrawal (Corrigan & Rao, 2012). Ultimately, self‐stigma related to mental illness can have negative consequences for wellbeing and negatively affect the recovery process (Mills et al., 2020).

Self‐stigma can negatively affect disclosure and help‐seeking for people living with mental illness. Additionally, there is a correlation between perceived stigma and negative help‐seeking attitudes for nurses (Yilmaz & Beydag, 2022), potentially impacting care delivery and leading to burnout (Bergman & Rushton, 2023). While nurses have higher health literacy and less challenging access to mental health support services, they do not necessarily seek appropriate health care when needed (Zaya et al., 2021). Alongside this, nurses have been found to report higher levels of adverse mental health outcomes, including depression or post‐traumatic stress disorder (PTSD) (Søvold et al., 2021). Nurses fear retribution in seeking mental health help, lack of support services, or the stigma that their ability to practice will be called into question (Southwick, 2022). This can result in burnout, compassion fatigue, and vicarious trauma, placing considerable challenges on the mental health workforce.

### 1.4 Self‐stigmatizing attitudes in nursing students

There is a recommendation to incorporate the measurement of self‐stigma in evaluating interventions that aim to decrease mental health related stigma in nursing education (Martin et al., 2020). As educational classroom‐based initiatives are not deemed effective in reducing stigma and self‐stigma for nursing students on their own (Fernandes et al., 2022), contact‐based interventions and the incorporation of lived experience education have been recommended as potentially combating undergraduate nursing student stigma toward mental illness and help‐seeking (Valentim et al., 2023). However, a dearth of research has sought to investigate the impact of contact‐based interventions on nursing student stigma in these domains. Foster et al. (2019) measured student nurses' stigmatizing attitudes before and after attending a traditional (i.e. hospital‐based) mental health clinical placement; though stigmatizing attitudes and desire to social distance were reduced post‐placement, student stigma surrounding disclosure and help‐seeking was unaffected. Investigating the impact of contact‐based interventions for undergraduate nurses' self‐stigma is burgeoning amidst the need to retain and protect the mental health of registered nurses.

### 1.5 Research aim

Pre‐registration nurse stigma toward mental illness, in the form of desire to social distance, is effectively reduced by attending a non‐traditional mental health clinical placement characterized by immersion alongside consumers living with enduring mental illness (Moxham et al., 2016). This non‐traditional model of clinical placement also significantly improves the mental health of consumers in measures of self‐determination (Taylor et al., 2017), motivation (Picton et al., 2018), hope (Moxham et al., 2015), and recovery (Patterson et al., 2023). However, it has not yet been assessed whether attending this model of mental health clinical placement can also reduce student nurse stigmatizing attitudes, and stigma surrounding disclosure/help‐seeking. The present study aimed to investigate whether attending a non‐traditional clinical placement effectively reduced three distinct types of pre‐registration nurses' stigma toward mental illness: stigmatizing attitudes, desire to social distance, and stigma surrounding disclosure/help‐seeking.

## 2 MATERIALS AND METHODS

### 2.1 Design

A prospective cohort design was used to test for changes in pre‐registration nurse stigma toward mental illness after attending a non‐traditional mental health clinical placement. Specifically, the effect of the placement was examined on three types of stigma: stigmatizing attitudes, desire to social distance, and stigma surrounding disclosure/help‐seeking.

### 2.2 Participants and setting

This study occurred in Australia, where pre‐registration nursing students require 800 clinical placement hours. For comparison, students in the United Kingdom require 2300 h of clinical placement, while in Australia, the number of hours required varies state by state (Roberts et al., 2019). In Australia, Recovery Camp is a mental health clinical placement contributing 80 clinical placement hours (see Molloy et al., 2020; Picton et al., 2018). This non‐traditional clinical learning experience is a five‐day, therapeutic recreation‐based program set amidst natural bushland at regional Australian sites (Molloy et al., 2020). Via the activities program (which can involve group activities such as archery, giant swing, dancing, rock climbing and craft), the placement promotes immersive social contact between students and people with lived experience of mental illness. It is facilitated by specialist mental health nurse educators, and students learn directly from experts with lived experience, such as those living with schizophrenia, bipolar affective disorder, suicidality, and PTSD. A cohort sample of Australian pre‐registration nursing students (*N* = 874, *M*_AGE_ = 28.43 years, SD = 9.09, 87.15% female) attended the mental health clinical placement as a part of their tertiary studies. The eligibility criteria for invitation to participate in the present study included current enrolment in either the second or third year of an Australian undergraduate nursing degree.

### 2.3 Ethical information

Ethical approval was granted by the institutional HREC panel (approval no: 2019/ETH03767) prior to commencing the study. Participants were given information prior to commencement, indicating that they had the option to withhold consent and/or withdraw from the study at any time, with no impact on their academic studies or their involvement in the clinical placement.

### 2.4 Measures

In addition to collecting demographic characteristics (gender, age), data were collected for each participant on the following:

#### 2.4.1 Opening minds scale for healthcare providers

The opening minds scale for healthcare providers (OMS‐HC) is a 15‐item self‐report scale validated for use across 32 countries amongst healthcare workers and students, social workers, nursing and medical students, child and adult psychiatrists, and general inpatient psychiatric ward staff (Őri et al., 2023). Items on the instrument are scored from 1 (strongly disagree) to 5 (strongly agree) and cluster into three specific factors measuring: (i) Attitudes toward people with mental illness (6‐items, e.g. ‘Despite my professional beliefs, I have negative reactions towards people who have mental illness’), (ii) Disclosure/help‐seeking attitudes (4‐items, e.g. ‘If I had a mental illness, I would tell my friends’) and (iii) Social distance (5‐items, e.g. ‘I would not want a person with a mental illness, even if it were appropriately managed, to work with children’). Items 2, 6, 7, 8 and 14 are reverse‐scored. Scores on the OMS‐HC can range from 5 to 75, with higher scores indicating higher stigma. An overall score can be summed across all items to index general stigma; scores can also be summed within each subscale outlined above to measure specific stigmatizing attitudes. Reliability analyses showed the OMS‐HC demonstrated an acceptable level of internal consistency at both the general (*α* = 0.79) and specific (*Attitudes α* = 0.71, *Social Distance* α = 0.70, *Disclosure*/*Help*‐*Seeking α* = 67) factor levels (Modgill et al., 2014).

### 2.5 Procedure

The OMS‐HC was deployed electronically via the survey platform Qualtrics at two time points: pre‐placement entry, and at exit. Eligible nursing students were invited to participate in the study via a Qualtrics email sent to their institutional email address. Each survey began by presenting information about the study aims, risks, and benefits, as well as the relevant ethical and confidentiality information. Students were advised that their participation was quasi‐anonymous through the use of a unique identifier created for each participant. The unique identifier served to tether the data observations between time points as well as de‐identify their survey responses. After this information was presented, informed consent was collected electronically.

Completion of the electronic surveys took approximately 10 min on each occasion. The pre‐placement survey was sent to all eligible nursing students 4 days prior to attending the placement. It could be completed using a personal computer or mobile device. The post‐placement survey was sent to students on the final morning of the clinical experience and could be completed at any time using a mobile device or personal computer. Participants could respond to the survey invitation at their own discretion during an administrative hour provided for students and consumers on the final morning of the clinical placement. Alternatively, the post‐placement survey could be completed at any time up to 7 days following the placement.

### 2.6 Data preparation and analysis

Data were collected between April, 2019 and April, 2024. In the first stage of data processing, all survey responses (*N* = 874) were exported from the Qualtrics platform and inspected for data quality in Excel. The response rate post‐placement was 83.5%. Responses with missing values (*n*_Pre_ = 26, *n*_Post_ = 15) were excluded from the analysis. The finalized data set comprised responses from 848 participants.

### 2.7 Statistical analysis

Pre‐ and post‐observations in the finalized dataset were analysed via a multivariate analysis of variance (MANOVA) using the SPSS statistical software package. The MANOVA tested for differences in each of the dependent variables (Attitudes, Social Distance, Disclosure/Help‐Seeking) in association with the factor of time (Pre, Post). Following any significant result, Bonferroni‐corrected post‐hoc comparisons were performed to determine the nature and strength of the difference for the relevant variable between time points.

A power analysis conducted in G*Power (v3.1) indicated a sample size of *N* = 106 would have 99% statistical power to detect moderate effects if using a repeated‐measures multivariate analysis of variance (within‐subjects, *f* ^2^ = 0.15, *α* = 0.05, 1‐β = 0.99) (Faul et al., 2009).

## 3 RESULTS

A MANOVA was used to test for a significant effect of attending the non‐traditional mental health placement on the stigma subscales of Attitudes, Social Distance, and Disclosure/Help‐Seeking. The analysis identified a significant multivariate effect of time on stigma scores, *F*(3, 702) = 26.21, *p* < .001, $\eta_{p}^{2}$= .101. This effect size can be considered large (Cohen, 1988). Gender was included to explore whether changes in stigma differed in relation to this factor; no effect was identified for any stigma domain (all *p* > .05).

**FIGURE 1:**
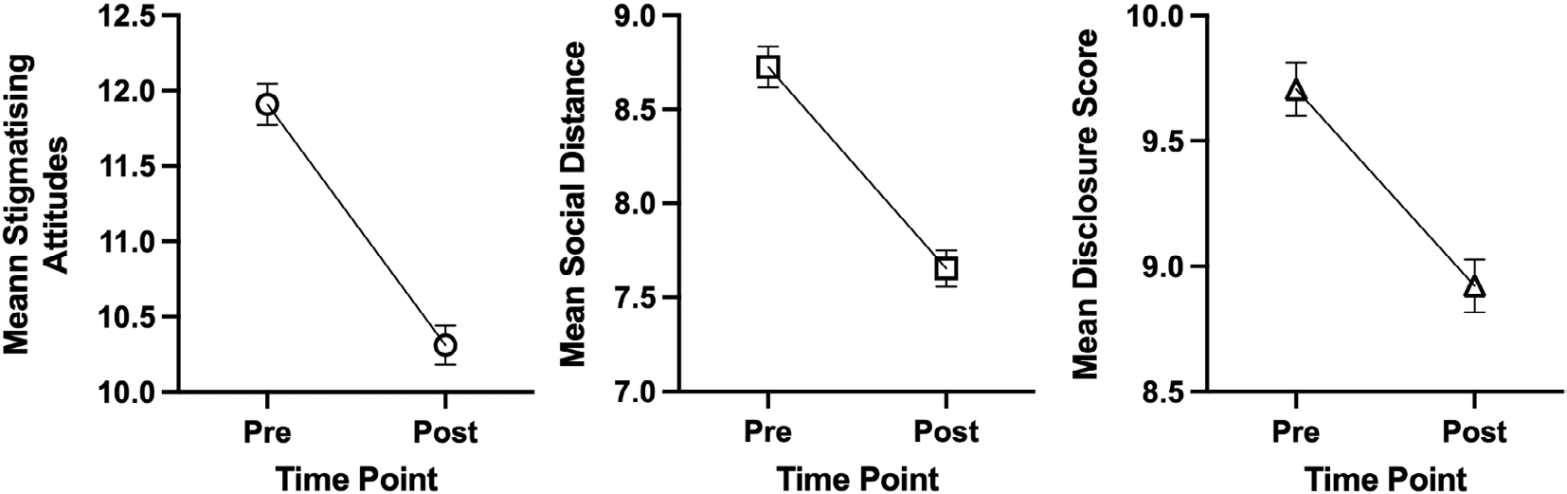
The stigmatizing beliefs of pre‐registration nursing students decreased in all three stigma domains after attending the non‐traditional clinical placement. Error bars indicate +/− 1 standard error.

Post hoc analyses showed that nursing students' stigma significantly decreased in all three domains after attending the mental health clinical placement: *Attitudes*: *F*(1,704) = 68.11, *p* < .001, $\eta_{p}^{2}$= .09, *Social Distance*: *F*(1,704) = 51.02, *p* < .001, $\eta_{p}^{2}$= .07, and *Disclosure/Help‐seeking*: *F*(1,704) = 27.39, *p* < .001, $\eta_{p}^{2}$= .04. These effect sizes can be considered moderate‐to‐large (Cohen, 1988). Each decrease in stigma is illustrated per stigma type in Figure 1.

## 4 DISCUSSION

The present study investigated whether a non‐traditional mental health clinical placement could effectively reduce nursing student stigma. Specifically, this study aimed to examine whether the non‐traditional placement could decrease nursing students' stigmatizing attitudes, desire to social distance, and stigma surrounding disclosure/help‐seeking. This aim was particularly motivated by a shortage of knowledge about clinical placement interventions and their impact on stigma, especially regarding disclosure and help‐seeking behaviours.

Our findings show that following the clinical placement, nursing student stigma significantly decreased in all three domains: attitudes, social distance and disclosure/help‐seeking. Regarding stigmatizing attitudes and social distance, these findings are consistent with those investigating nursing student stigma following mental health clinical placements (Foster et al., 2019; Happell, 2008; Henderson et al., 2007; Moxham et al., 2016). However, the present study is novel in finding the effectiveness of a non‐traditional clinical placement in reducing all three aspects of stigma; notably, in the disclosure/help‐seeking domain.

Thus far, the effect of mental health educational initiatives has seen improvements in help‐seeking intention (Picton et al., 2018). Yet knowledge of the impact of a clinical placement intervention on self‐stigma remains scarce. Martin et al. (2020), investigated the impact of a preclinical psychiatry course, recognizing a link between improving student knowledge on mental illness and an improvement in self‐stigma and help‐seeking attitudes. Going forward, the use of instruments that examine and measure self‐stigma, is useful in evaluating interventions that improve help‐seeking for pre‐registration student nurses (Martin et al., 2020). Foster et al. (2019) found no improvements in stigma related to disclosure/help‐seeking following a traditional clinical placement. However, aspects of a non‐traditional placement that are conducive to reduced stigma, as found in this study, are of interest. One possible reason for reduced stigma in association with disclosure and help‐seeking may be the immersive nature of the non‐traditional clinical placement. An immersive placement, such as the one used in this study, is designed to increase the exposure and educative‐connection between nursing students and people living with mental illness (Molloy et al., 2020; Picton et al., 2018).

Positive mental health clinical placements play a major role in pre‐registrations nurses' decisions to pursue psychiatric nursing. Increasing contact with people living with mental illness through clinical placement and consumer‐led education, is seen to improve nursing student attitudes toward people living with mental illness (Happell, 2008). Personal and prolonged contact with people living with mental illness has been well documented to positively influence stigma and negative attitudes toward mental illness (Happell, 2008; Knaak et al., 2017; Perlman et al., 2020). Tratnack et al. (2011) study on brief clinical immersion for pre‐registration nursing students and people living with mental illness, found that students were better able to develop clinical skills and build therapeutic relationships, urging further immersion to happen in mental health nursing placement settings. One theory that attempts to explain the benefits of prolonged immersion is that of intergroup contact theory (Pettigrew & Tropp, 2011). Intergroup contact theory (ICT) purports that face‐to‐face contact with people belonging to a different group decreases prejudice, perceptions of threat, and stigma (Allport, 1954; Pettigrew & Tropp, 2011). Outside of the clinical placement setting for pre‐registration nurses, interventions that have utilized ICT including people living with mental illness, have found a significant reduction in self‐stigma for participants without mental illness (Evans‐Lacko et al., 2012; Martínez‐Hidalgo et al., 2018; Maunder & White, 2019).

Recovery Camp is an immersive, non‐traditional clinical placement that has utilized the principles of ICT to reduce stigma (Moxham et al., 2015) and nursing clinical confidence (Patterson et al., 2017). Thus far, non‐traditional clinical placements have resulted in increased confidence in working with consumers (Stuhlmiller, 2003), and increased self‐esteem (Stuhlmiller, 2003). Happell et al. (2015) suggest that a well‐supported and person‐centred clinical placement is vital in improving the recruitment of mental health nurses. A key part of this process is reducing the stigma surrounding disclosure and help‐seeking, which has become a priority for nurses amidst the increased stressors experienced following COVID‐19, recognizing a need to encourage help‐seeking behaviours and reduce burnout (Mannix, 2021). With a range of factors affecting burnout in nurses (Dall'Ora et al., 2020), improving help‐seeking attitudes in pre‐registration nursing students is paramount to improve the attrition and retention of mental health nurses.

### 4.1 Limitations

This study has several limitations. While we can show that nursing students' stigma regarding attitudes, desire to social distance, and disclosure and help‐seeking was reduced after the non‐traditional mental health clinical placement, the factors influencing this finding are unclear. Further research in this area could focus on which attributes of the clinical placement setting foster positive help‐seeking. Second, the students in this study reside in Australia, potentially influencing the generalizability of these results to nursing students in other countries. Third, the non‐traditional clinical placement environment used in this study is unique, and further research is warranted regarding which specific aspects of the environment may be influencing stigma. The lack of a comparison group restricts our consideration of the placement features that may be facilitating these effects, and is noted as a potential limiting factor.

### 4.2 Recommendations for practice

The results of this study outline the benefits of a non‐traditional mental health clinical placement in reducing self‐stigma for student nurses, with a dearth of research measuring self‐stigma in the pre‐registration nursing student population before and after a clinical placement. Further research could focus on which aspects of the placement environment effectively decreased nursing students' stigma in relation to disclosure and help‐seeking. It would also be worthwhile for future studies to explore how this may longitudinally affect disclosure and help‐seeking in this population.

### 4.3 Conclusion

Stigma toward mental illness causes major barriers to people seeking healthcare, negatively impacts the care given by healthcare professionals, and contributes to the lower numbers of students to pursue mental health nursing upon registration. Additionally, self‐stigma is a major barrier to nurses seeking their own mental health support and reducing burnout. Upon engagement with a non‐traditional mental health clinical placement, pre‐registration nursing students experienced decreases in the three domains of stigma as measured by the OMS‐HC, including attitudes, social distance, and disclosure and help‐seeking. This is one of the only studies to find a significant decrease in self‐stigma following a mental health clinical placement for student nurses. Additionally, this study shows there is potential for clinical placements to enhance nursing students' mental health disclosure and help‐seeking. Further research is required to investigate the specific aspects of the non‐traditional clinical placement environment that can reduce self‐stigma and encourage disclosure and help‐seeking behaviours—And how these may be translated to traditional settings. Improving stigma surrounding disclosure and help‐seeking via clinical placements may contribute to better care for individuals with mental illness and support the long‐term health of mental health nurses.

## 5 RELEVANCE STATEMENT

Stigma amongst nurses negatively impacts help‐seeking attitudes and impacts patient care. While clinical placement interventions have been effective in reducing stigma related to attitudes and social distance, there are nil recorded interventions that decrease stigma surrounding disclosure and help‐seeking in relation to mental health. The present study investigates the effect of a non‐traditional mental health placement on pre‐registration nurse's stigma in the domains of attitudes, social distance, and disclosure/help‐seeking. The results provide valuable information about how to improve help‐seeking attitudes in the aim to prevent possible burnout.

## AUTHOR CONTRIBUTIONS

**Christopher Patterson**: conceptualization, methodology, reviewing and editing. **Michelle Roberts**: methodology, software, data curation, formal analysis, writing—original draft preparation. **Taylor Yousiph**: software, data curation, formal analysis, writing—original draft preparation. **Georgia Robson**: software, data curation. **Kelly Lewer**: writing—reviewing and editing. **Elissa‐Kate Jay**: writing—reviewing and editing. **Lorna Moxham**: supervision, conceptualization, methodology, reviewing and editing.

## CONFLICT OF INTEREST STATEMENT

Recovery Camp is a research and social impact program that has been supported by the University of Wollongong to explore its potential as a social enterprise. Authors C. Patterson and L. Moxham are directors of Recovery Camp Pty. Ltd.

## ETHICS STATEMENT

Ethical approval was obtained from the relevant institutional ethical board (approval no: 2019/ETH03767).

## Data Availability

The data that support the findings of this study are available from the corresponding author upon reasonable request.
